# Ask a Doctor a Question: A Clinician’s Message to the Industry

**DOI:** 10.3390/medicina61030368

**Published:** 2025-02-20

**Authors:** Audrius Andrijauskas, Povilas Andrijauskas, Ieva Jovaišienė, Arūnas Valaika, Tomas Jovaisa, Karolis Urbonas, Darius Činčikas, Saule Svediene, Nadezda Scupakova, Lina Puodziukaite, Mindaugas Budra, Gintaras Kalinauskas, Edgaras Stankevičius

**Affiliations:** 1 Clinic of Anaesthesiology and Intensive Care, Institute of Clinical Medicine, Faculty of Medicine, Vilnius University, 01513 Vilnius, Lithuania; audrius.andrijauskas@mf.vu.lt (A.A.); ieva.jovaisiene@santa.lt (I.J.); tomas.jovaisa@santa.lt (T.J.); dariuscincikas@yahoo.com (D.Č.); saule.svediene@mf.vu.lt (S.S.); lina.puodziukaite@santa.lt (L.P.); 2 II Department of Anaesthesiology and Intensive Care, Vilnius University Hospital Santaros Klinikos, 08661 Vilnius, Lithuania; karolis.urbonas@santa.lt (K.U.); nadezda.scupakova@santa.lt (N.S.); 3 Clinic of Cardiac and Vascular Diseases, Institute of Clinical Medicine, Faculty of Medicine, Vilnius University, 01513 Vilnius, Lithuania; arunas.valaika@santa.lt (A.V.); mindaugas.budra@santa.lt (M.B.); gintaras.kalinauskas@santa.lt (G.K.); 4 Institute of Physiology and Pharmacology, Lithuanian University of Health Sciences, 44307 Kaunas, Lithuania; edgaras.stankevicius@lsmu.lt

**Keywords:** non-invasive, monitoring, PPG, oedema, tissues, fluid, measure, anaesthesia, intensive care, industry, physician

## Abstract

The medical industry is an integral part of the delivery of healthcare. Collaboration between academic institutions, healthcare providers, and the industry are necessary but not devoid of flaws. This expert opinion article calls for closer attention to be paid by the medical industry to “what a frontline clinician needs” rather than relying solely on experts’ opinions and stake holders’ requests in planning future products and features. The need for the monitoring of tissue fluid accumulation is discussed from the point of view of practicing anaesthesiology and intensive care specialists in the context of the potential missed opportunity to have it be already available.

## 1. Introduction

Physicians and the industry have a common interest in advancing medical knowledge. Physicians attend professional meetings with pharmaceutical representatives and participate in the research and development of industrial products [1]. However, the physician’s primary goal is to promote their patient’s interests, while the industry’s is marketing products, which are not necessarily specifically targeting the benefit of the patient [2]. Therefore, these interactions can create conflicts of interest that affect clinical judgments, prescription, research, education, and treatment outcomes [2,3].

The marketing divisions of pharmaceutical and medical device firms view clinicians as the main targets of their product promotion efforts [4]. Thus, hospital visits by industry representatives are dedicated to promoting their product, providing training, and receiving feedback about the experience of using it. Industry representatives use a variety of promotional methods including leaflets, drug samples, and free “test rides” of promoted devices [5]. However, product development divisions’ representatives do not show up to find out what the frontline doctors’ expectations are for future products. That is where the missed opportunity to develop a very much needed product can occur.

It is not unusual for doctors to take jobs in the industry where they work closely with basic scientists to design studies and clinical trials. However, usually they are not the ones who propose major innovations or define future trends in product development. The vision of future product development mainly relies on recommendations from academic institutions and contract research organisations. Publications and presentations at scientific events are the other sources of obtaining insights about the healthcare providers’ expectations and the possible ways to pursue them. However, to the best knowledge of the authors, there have been no scientific meetings that have had a section such as “ask a doctor a question”, where on-site and online attendants—practising clinicians—could have been asked what they want from future medical products or what ideas they have about how to improve the state-of-the-art products they are using.

This expert opinion article briefly addresses the pitfalls related to potential conflicts of interest in the relationship between the medical industry and healthcare stakeholders, academic institutions, and clinicians, and calls for a more end-user centred approach in planning future products.

Research and development is the bedrock of innovation, with significant investments in this area [6,7,8]. This article advocates that frontline clinicians should be more involved in initiating research projects and proposing innovative ideas rather than being used as data collectors. Not paying attention to justified on-site clinical needs sets the case for the missed opportunity for industry to develop, in a cost-effective way, a very much needed new product, when a similar technique is being developed for other purposes.

As an example, an obvious need for clinically practical technology to monitor fluid accumulation is briefly reviewed in the context of a potentially missed opportunity to develop such a device when the relevant spectrophotometric techniques were developed for other purposes.

## 2. Pitfalls in Collaboration Between Medical Professionals and Industry

The health sector is a dynamic system composed of complex interactions between patients, providers, payers, suppliers, and policy makers. It is exactly this complexity that makes it particularly vulnerable to corruption [9]. A recent study in JAMA [10] finds that 57% of physicians have received a payment from pharmaceutical or medical device companies over the past ten years.

The Physician Payments Sunshine Act led to the creation of the Open Payments US database, a repository of industry payments to healthcare professionals. The JAMA study [10] examined industry payments to physicians through that portal, looking at payments made between 2013 and 2022, not including research grants. In total, drug and device companies paid about $12 billion to more than 800,000 physicians during this period [10]. The median payment for doctors was $48, but some doctors were paid in the millions. For example, the average amount paid to the top 0.1% of orthopaedic surgeons was over $4.8 million.

There is a long tradition of collaboration between medical professionals and the industry in the interests of medical progress [11]. Physicians attend professional meetings with industry representatives and participate in research, development, and investment for health-related industries. Nevertheless, the possible bias in opinion formation is concerning [12]. Thus, publishing industry-sponsored studies are under scrutinised review [13].

Medical devices entail an even greater degree of physician–industry interaction regarding treatment, training, and innovation compared to pharmaceuticals. Bergman et al. [14] has summarised and compared device and drug firm payment rates and magnitudes. The total spending in the US prescription drug market between 2014 and 2017 was $1378 billion [15], while the size of the implantable medical device market was $211.3 billion [16].

Medical device industry payments differ from pharmaceutical industry payments. Medical device companies are paying physicians more for product development (royalties, licencing, and investment) and education, while pharma companies are paying more for speaking fees and food and beverage costs.

Despite evidence that conflicts of interest may influence decision-making and damage patients’ trust in professionals [17], such financial relationships remain pervasive [18]. What are the implications of these industry payments? Research indicates that physicians are much more likely to use a device made by the manufacturer that gives them the largest payment [18]. For doctors that hold positions on panels and decision-making bodies, conflicts of interest can influence guidelines, policies, as well as orders from the industry.

A discussion of the possible impact of conflicts of interest on product development priorities is not within the scope of this article. What has been discussed serves as context to the next section, where the main message is that every frontline clinician devoid of any conflicts of interest is a valuable unbiased source of information regarding expectations for new features and devices that might have a potential to improve patient care and rationalise the workload of care providers. There are many examples when even innovative ideas came from doctors without academic affiliations.

## 3. Missed Opportunity?

Next, we discuss the need for clinically practical technology for monitoring fluid accumulation in body compartments, and a potentially missed opportunity to develop such a technology when spectrophotometric techniques were developed for other purposes, e.g., non-invasive haemoglobin. Firstly, the necessity of such technology and the feasibility of its development is briefly discussed without a literature review or in-depth analysis, with an aim to illustrate how it could be presented by a non-academic clinician.

Following the dictum “primum non nocere” [19], the prevention of treatment-related adverse events is non-inferior to an objective of improving patients’ health [20]. Complications with treatment have a huge impact on morbidity, mortality [21], and healthcare costs [22]. Delivering care based on a so-called risk-adapted approach is essential to enhance healthcare value in the face of rising demands and resource constraints [23]. Ensuring adequate tissue perfusion and oxygenation is considered the ‘‘holy grail’’ of cardiovascular management in fluid therapy [24,25]. The adverse effects of infusion fluids are associated with worse outcomes [26]. The prevention of oedema, a notorious adverse effect, is an important component of patient-focused care with an aim to optimise the quality and cost of care.

Almost all but the most minor in-hospital procedures begin with an intravenous fluid infusion. It is such a routine part of clinical practice that few give any serious thought as to how is it titrated and how safe it is. Fluids are infused to maintain, restore, or elevate the blood volume by diluting plasma. As the plasma volume increases, water and electrolytes pass out of circulation into the tissues. In healthy individuals, as the volume expansion of the plasma is reversed by renal excretion, fluid passes back from the interstitium into the plasma, maintaining homeostasis.

Healthy individuals can cope with a wide range of infusion volume and accumulation levels in circulation and tissues, but critically ill individuals and patients undergoing major surgery are extremely vulnerable. Another major difference between the healthy and the sick is that thirst is a reliable indicator of the need for fluid only in the healthy. In clinical settings, assessing the need for fluid or fluid restriction or removal is based on clinical signs and indirect estimates, which are not sufficiently sensitive to provide warning of imminent deteriorations in hydration—oedema or dehydration. A major flaw in the clinical interpretation of fluid balance is the missing criteria for discrimination between necessary and detrimental fluid accumulation.

Since clinical fluid management is mainly guided by invasively and non-invasively monitored hemodynamic responsiveness, estimates of fluid balance, and invasive parameters such as extravascular lung water for detecting the life threatening accumulation of fluid in the lungs, adverse effects of fluid therapy, and oedema in particular, are not uncommon.

## 4. Oedema

During human evolution, the body has been more exposed to a lack of water rather than an abundance, and thus has become resilient to dehydration, but vulnerable to overhydration. Fluid accumulation in circulation and tissues is an inherent side effect [27]. It turns into an adverse event when it overloads circulation (hypervolemia) and/or tissues (oedema) [28]. There are reliable methods for minimising the risk of circulation overload-related adverse events [29], but there is no clinically useful method for quantifying interstitial fluid accumulation [24] and preventing oedema [26]. Under the existing methods, oedema is detected in its late stages [30]. Eventually, oedema causes organ dysfunction, to the point of causing death [26].

Oedema is an independent predictor of hypoperfusion, hypoxia, and organ dysfunction [31,32,33]. It can develop fast and unnoticed, e.g., during fluid challenges, and especially in conditions such as sepsis with increased capillary permeability due to glycocalyx shedding [34,35]. Oedema can occur from both crystalloids and colloids [36]. Crystalloids are the most frequently used fluids [31,35,37]. They are distributed with a preferential interstitial allocation of the overload to the skin, gastrointestinal tract, and lungs, with potentially dangerous consequences, e.g., swelling of the intestinal wall leads to an anastomotic leak and the delayed recovery of intestinal mobility [38].

Oedema after fluid resuscitation protocols is common and fluid removal is frequently necessary [39]. However, insufficient or excess fluid removal related to the conventional rule-of-thumb administration of intravenous diuretics often occurs and brings new complications [40]. Assessing the efficacy of fluid removal is based on the evaluation of urine output, changes in cumulative fluid balance, a reduction in signs and symptoms of fluid overload such as the pitting oedema of peripheral tissues, and pulmonary oedema. However, fluid removal may cause unnecessary dehydration because the above-mentioned tools lack sensitivity and specificity in the quantitative determination of interstitial fluid levels and the detection of imminent dehydration. Individualised fluid removal could improve the safety of fluid removal, given that more reliable technology for monitoring transcapillary fluid shifts and changes in fluid accumulation becomes available [41,42].

Chronic fluid overload is associated with morbidity and mortality in haemodialysis patients. For decades, developing reliable methods for the diagnosis and treatment of fluid overload has remained a priority in nephrology, and recent advances in the development of wearable applications are promising [43,44]. However, aside from differences between the pathophysiology of fluid accumulation and approaches in fluid removal during fluid therapy and renal failure settings, the main limitation in bioimpedance techniques is the inability to discriminate between intravascular and extravascular fluid volumes, since they measure extracellular and total body water. Since bioimpedance is limited to monitoring transcellular fluid shifts, the transcapillary fluid equilibration and the fluid accumulation specifically in circulation and tissues cannot be monitored, and thus, fluid requirements cannot be reliably assessed during fluid therapy. A combination of transthoracic bioimpedance and heart rate variability measurements with a wearable vest are used to monitor fluid accumulation in lungs and detect acute decompensated heart failure [45]. However, this might serve as a diagnostic tool to detect the adverse effects of fluid therapy, rather than to prevent them or to guide fluid management.

Microcirculation is where oxygen is delivered and consumed [46]. Organ dysfunction due to oedema occurs via the deterioration of microcirculation. Oedema causes hypoxia by compressing capillaries, as well as increasing the distance that oxygen molecules have to travel from the capillary to the cell [24]. Thus, it would be reasonable to use microcirculatory monitoring as a primary tool to titrate fluids [28,47]. Side stream dark field (SDF) [48] and incident dark field (IDF) video microscopy, Laser Doppler velocimetry (LDV) [49], Skin Laser Doppler (SLD) [50], near-infrared spectroscopy (NIRS) [51], thermography [52], and Laser Speckle Imaging (LSI) [53] are common techniques. However, they mainly serve as tools for research [53,54]. Parameters such as total vessel density (TVD) seem to be attractive endpoints for fluid administration [55] and monitoring tissue fluid accumulation, but the inability of the technology to provide automated continuous real-time quantitative evaluation and analysis of alterations is a major limitation [53].

Endothelial glycocalyx, a protective carbohydrate-rich layer lining the luminal surface of the endothelium, plays a key role in vascular barrier dysfunction, oedema formation, and organ failure [56]. Glycocalyx degradation (shedding) is related to a stress response, ischemia–reperfusion injury, systemic inflammatory response, and sepsis. The detection of shedding is an alert about an increased risk of oedema. The diagnosis is based on the amount of shed constituents, such as syndecan-1, in serum [57,58]. However, increased levels [59] do not provide information about the extent of glycocalyx damage [60,61]. The measuring of levels is not continuous and thus has limited value for the titration of fluids and oedema risk assessment during fluid therapy [62,63,64,65]. The microscopic measurement of perfused boundary regions is used for the evaluation of glycocalyx thickness [57,66], but it is not clinically practical—it is cumbersome, imprecise, and retrospective.

There are three “pillows” of cardiovascular resuscitation: (1) maintaining adequate circulating blood volume and circulatory pressure (intravenous fluids and cardiovascular medication), (2) avoiding the side effects of fluid therapy such as fluid overload and oedema (monitoring fluid accumulation and detecting imminent oedema), and (3) ensuring adequate oxygen delivery to the organs and cells (maintaining adequate haemoglobin levels and the perfusion of microcirculation). The monitoring of fluid accumulation in tissues is a major missing technology.

This brief review shows the obvious clinical necessity for monitoring tissue fluid accumulation with an aim to provide safe and rational fluid therapy measures.

## 5. A Message to Industry

The fluid shift between the blood circulation and tissues is an essential mechanism of homeostasis. The net fluid shift is determined by the net transcapillary fluid equilibration occurring across the body’s network of microvasculature.

The processes that guide transcapillary fluid equilibration are extremely complex [67]. Theories and models are continuously developed [67,68]. Interstitial fluid accumulation and oedema formation mechanisms have remained the focus of research for decades [69,70,71]. Given the undeniable complexity that underlies fluid shift between the intravascular and tissue compartments, it is feasible to monitor the net effect of the confounding factors—the fluid levels in tissues. Assuming that it would be extremely difficult to determine the individually optimal levels, the trend-monitoring approach could be a rational approach for assessing the dynamics of fluid accumulation. Is it technologically “mission impossible” or a “missed opportunity” by the industry? In the next chapter, this aspect is explored in the context of modern optical spectroscopy from the point of view of non-academic clinicians in surgery, anaesthesiology, and intensive care.

## 6. Spectrophotometry

Spectrophotometry is one of the most useful methods of quantitative analysis in various fields such as chemistry, physics, biochemistry, material and chemical engineering, and clinical applications. At the beginning of 21st century, researchers started to work on non-invasive blood constituent monitoring by exploiting the wavelength dependence of light penetration in tissues to measure light absorption by specific constituents. As highlighted in a recent review [72], advances in multi-wavelength (MW) photoplethysmography (PPG) have led to unprecedented progress in non-invasive monitoring. Diffuse reflectance spectroscopy is widely used for general and medical applications to analyse the optical characteristics of biological tissues [73].

The famous clinical application of MW PPG is a pulse oximetry technique for measuring arterial oxygen saturation (SpO_2_). Non-invasive technology—pulse CO-oximetry—for the continuous measuring of total haemoglobin (SpHb; Masimo Corp., Irvine, CA, USA) has yielded some of the most groundbreaking results in haemoglobin trend monitoring [72]. Masimo W1^®^ Medical is the first FDA-cleared medical wearable to provide continuous, real-time oxygen saturation (SpO_2_) and pulse rate (PR) information. Apple Watch has gained FDA 510(k) class II clearance for the ECG feature and ability to detect arrhythmias. Empatica Embrace 2 gained FDA 510(k) clearance for epilepsy detection. Machine Learning (ML) techniques are already used for exploiting the characteristic features of PPG signals [74] for monitoring, screening/diagnostic, decision support, and predictive purposes [75].

Since a myriad of parameters are measurable by MW PPG technologies, what are the odds of developing the reliable trend-monitoring of tissue fluid levels and estimates of fluid requirements? The commercially available Masimo W1 Watch incorporates over 30 years of Signal Extraction Technology pulse oximetry knowledge. It provides the Hydration index (Hi) as an indication of fluid status. However, the Hi is displayed in a dimensionless scale without the indication of deteriorations—oedema or dehydration. Moreover, the Hi is not provided by its most recent upgrade—the Masimo W1^®^ Medical Watch [76], which is the first FDA-cleared medical wearable.

## 7. Clinicians’ Insights

As already mentioned, fluid overload is preferentially allocated to the skin, gastrointestinal tract, and lungs. The skin is the largest organ, which contributes to about 16% of the body weight. The derma is the major site of high fluid compliance in the interstitium that can accumulate large amounts of fluid. Since net intravascular space has a limited ability to adapt to changing intravascular volume (blood volume), the first line of defence against the overstretching of circulation by acute excessive intravascular fluid load is “draining” it into the derma, because urinary elimination, if intact, requires up to 20–30 min to gain the necessary efficacy.

The derma is a fluid depot for the on-demand release of fluid to circulation for delivery to the vital organs that have a negligible ability to store fluid reserves. Meanwhile, it takes time for the thirst-triggered ingested water to enter the circulation. Thus, monitoring fluid levels in the derma would be a valuable tool for the general and clinical evaluation of fluid requirements, as well as transcapillary fluid shifts, given that simultaneous haemodilution monitoring is available. Is it feasible?

The MW PPG measurements are usually performed in the microcirculation of the skin. The evolution of techniques to assess skin hydration was recently reviewed [77]. The main light-absorbing components within the skin are water, melanin, and haemoglobin [78]. Each absorb light differently depending on the wavelength of light and chemical bonding. As reviewed above, haemoglobin and water are measurable using existing technologies, and thus their reliable trends should be available. Since it seems very unlikely that technological flaws are to blame for the unacceptable accuracy of estimates, is it the conceptual approach that needs to be revised?

The in vitro investigation of digital multi-wavelength optical sensors for the continuous non-invasive measurement of dermal water contention in porcine skin has shown acceptable accuracy [79]. Furthermore, the multi-modal assessment of skin hydration, employing both electrical and optical sensing modalities, has enhanced skin hydration measurement sensitivity and validity in ex vivo experiments on fresh porcine skin, and in an in vivo indicative case study [80]. What are the possible obstacles to replicating the accuracy results in a living human skin?

PPG is an optical technique which exploits the light which is shone through tissues, and the detected light intensity forms an electric signal—a photoplethysmogram. The PPG signal is composed of a pulsatile AC component and a non-pulsatile DC component. The DC component is underutilised when compared to the AC, but it is well documented that this slowly varying component is a carrier of valuable physiological information. Potentially, it can provide clinically useful information about the trend of tissue fluid accumulation extrapolated from measuring the trend of light attenuation by water in tissues at high wavelengths. Trending could minimise the impact of light absorption by constituents that have a constant volume. However, estimates of water based on the signal of DC component include intravascular volume (plasma), intracellular fluid in red cells, extravascular interstitial water, and intracellular fluid too. Thus, if tissues are compressed to the point of full obstruction of flow within the penetration of PPG light, only the extravascular fluid volume would be absorbing light. Repeatedly performed occlusion sessions would provide a trend of fluid accumulation in tissues. A difference between the DC signal pre- and post-occlusion would indicate the volume of intravascular fluid which would serve in monitoring the trends of intravascular fluid accumulation and haemodilution. The latter would be clinically valuable for the evaluation of the volume effects of infused fluids, as well as removed fluids. Is this common sense?

There is a strong correlation between fluid accumulation in the skin and skeletal muscles since they have a similar interstitial fluid compliance [69,81]. Thus, monitoring fluid accumulation trends in skin can indicate a similar tendency in muscles, which, taken together, comprise the majority of the fluid accumulation capacity of the body. Can the whole-body fluid status be determined?

## 8. Implications

The technology for monitoring fluid accumulation could have far-reaching repercussions, finding application in general practice-based primary care, emergency, trauma, resuscitation, and intensive care; pre-, peri-, and post-operative care; and domiciliary healthcare. Such a variable, in parallel to currently available estimates, would have the potential to become as ubiquitous as a thermometer is today for helping to diagnose anaemia, blood loss, and fluid requirements.

In the evolving landscape of space exploration, the need for advanced medical care during extended missions to the Moon and Mars becomes a critical challenge. The risks associated with trauma, haemorrhagic shock, infections, and physiological changes due to microgravity highlight the necessity for innovative medical care in remote, resource-constrained environments. Fluid distribution between the circulation and tissues in microgravity may be significantly affected by the redistribution of blood volume within circulation, as well as environmental factors such as exposure to ionising radiation and cardiac dysfunction [82,83]. Moreover, ionising radiation may damage the capillary endothelial layer, which is the capillary barrier [84]. These three factors are the “perfect storm” for causing the transcapillary leak of fluids into the tissues and causing oedema with the consequent life-threatening deterioration of microcirculation and oxygen delivery to cells. Monitoring transcapillary fluid distribution and accumulation is vitally important for providing fluid therapy and cardiovascular rescue measures in microgravity.

## 9. Conclusions

There is a justified need for a clinically practical continuous and non-invasive technology for monitoring fluid accumulation in tissues, with the aim of guiding fluid administration and removal. Among so many physiological processes in the vicinity of microcirculation that have gained the attention of the monitoring technique developers, the development of new conceptual approaches to the assessment of transcapillary fluid shifts and fluid accumulation seems to have been left in the shadows.

This article calls for closer collaboration between non-academic clinicians and the industry in planning and developing future clinical devices and their features. Just “ask a doctor a question—What do you want from tomorrow’s tech? Do you have ideas of how to develop it?”. Care providers are already overwhelmed by the abundance of parameters and monitors. Asking the question “What parameters that are not useful, and thus could be removed” is also relevant, e.g., it would be interesting to ask whether perfusion index (PI) is really useful in diagnostics and decision-making, or just occupies precious space on the monitors’ screen. Communication is vital at all levels.

## Data Availability

Not applicable.

## References

[1] Zarei E., Ghaffari A., Nikoobar A., Bastami S., Hamdghaddari H. (2023). Interaction between physicians and the pharmaceutical industry: A scoping review for developing a policy brief. Front. Public Health.

[2] Garattini L., Padula A. (2019). Conflict of interest disclosure: Striking a balance?. Eur. J. Health Econ..

[3] Stoll M., Hubenschmid L., Koch C., Lieb K., Egloff B. (2022). Physicians’ attitudes towards disclosure of payments from pharmaceutical companies in a nationwide voluntary transparency database: A cross-sectional survey. BMJ Open.

[4] Diekema D.S. (2022). Health Care Clinicians and Product Promotion by Industry. Pediatrics.

[5] Schramm J., Andersen M., Vach K., Kragstrup J., Peter Kampmann J., Søndergaard J. (2007). Promotional methods used by representatives of drug companies: A prospective survey in general practice. Scand. J. Prim. Health Care.

[6] Main Science and Technology Indicators (MSTI Database). https://www.oecd.org/en/data/datasets/main-science-and-technology-indicators.html.

[7] Latest Medical Research Funding Opportunities. https://www.myresearchconnect.com/funding-highlights/funds/medical-research/.

[8] Estimates of Funding for Various Research, Condition, and Disease Categories (RCDC). https://report.nih.gov/funding/categorical-spending#/.

[9] Glynn E.H. (2022). Corruption in the health sector: A problem in need of a systems-thinking approach. Front. Public Health.

[10] Sayed A., Ross J.S., Mandrola J., Lehmann L.S., Foy A.J. (2024). Industry Payments to US Physicians by Specialty and Product Type. JAMA.

[11] Jacob N.T. (2018). Drug promotion practices: A review. Br. J. Clin. Pharmacol..

[12] Chimonas S., Mamoor M., Zimbalist S.A., Barrow B., Bach P.B., Korenstein D. (2021). Mapping conflict of interests: Scoping review. BMJ.

[13] Coyle S.L., Ethics and Human Rights Committee, American College of Physicians-American Society of Internal Medicine (2002). Physician–Industry Relations. Part 1: Individual Physicians. Ann. Intern. Med..

[14] Bergman A., Grennan M., Swanson A. (2021). Medical Device Firm Payments to Physicians Exceed What Drug Companies Pay Physicians, Target Surgical Specialists. Health Aff..

[15] Hartman M., Martin A.B., Benson J., Catlin A. (2020). National Health Care Spending in 2018: Growth Driven by Accelerations in Medicare and Private Insurance Spending. Health Aff..

[16] BMI Research Industry Forecast—United States Medical Devices Forecast. https://www.fitchsolutions.com/bmi.

[17] Annapureddy A.R., Henien S., Wang Y., Minges K.E., Ross J.S., Spatz E.S., Desai N.R., Peterson P.N., Masoudi F.A., Curtis J.P. (2020). Association Between Industry Payments to Physicians and Device Selection in ICD Implantation. JAMA.

[18] Marshall D.C., Tarras E.S., Rosenzweig K., Korenstein D., Chimonas S. (2020). Trends in Industry Payments to Physicians in the United States from 2014 to 2018. JAMA.

[19] Gillon R. (1985). “Primum non nocere” and the principle of non-maleficence. Br. Med. J. (Clin. Res. Ed.).

[20] Rehberg S., Aken H.V., Westphal M. (2014). Fluid resuscitation in the 21st century: Don’t try to run before you are able to walk. Best Pract. Res. Clin. Anaesthesiol..

[21] Khuri S.F., Henderson W.G., DePalma R.G., Mosca C., Healey N.A., Kumbhani D.J. (2005). Determinants of long-term survival after major surgery and the adverse effect of postoperative complications. Ann. Surg..

[22] Shin C.H., Long D.R., McLean D., Grabitz S.D., Ladha K., Timm F.P., Thevathasan T., Pieretti A., Ferrone C., Hoeft A. (2018). Effects of Intraoperative Fluid Management on Postoperative Outcomes: A Hospital Registry Study. Ann. Surg..

[23] Grocott M.P.W., Edwards M., Mythen M.G., Aronson S. (2019). Peri-operative care pathways: Re-engineering care to achieve the ’triple aim’. Anaesthesia.

[24] Svensen C. (2016). Monitoring micro-vascular reactivity: A tool for guiding fluid therapy?. Anaesthesia.

[25] Scheeren T.W. (2016). Journal of Clinical Monitoring and Computing 2015 end of year summary: Tissue oxygenation and microcirculation. J. Clin. Monit. Comput..

[26] Hahn R.G., Hahn R.G. (2016). Adverse effects of infusion fluids. Clinical Fluid Therapy in the Perioperative Setting.

[27] Hahn R.G. (2017). Changing practices of fluid therapy. Acta Anaesthesiol. Scand..

[28] Chawla L.S., Ince C., Chappell D., Gan T.J., Kellum J.A., Mythen M., Shaw A.D. (2014). Vascular content, tone, integrity, and haemodynamics for guiding fluid therapy: A conceptual approach. Br. J. Anaesth..

[29] Chong M.A., Wang Y., Berbenetz N.M., McConachie I. (2018). Does goal-directed haemodynamic and fluid therapy improve peri-operative outcomes?: A systematic review and meta-analysis. Eur. J. Anaesthesiol..

[30] Arieff A.I. (1999). Fatal postoperative pulmonary edema: Pathogenesis and literature review. Chest.

[31] Feldheiser A., Aziz O., Baldini G., Cox B.P., Fearon K.C., Feldman L.S., Gan T.J., Kennedy R.H., Ljungqvist O., Lobo D.N. (2016). Enhanced Recovery After Surgery (ERAS) for gastrointestinal surgery, part 2: Consensus statement for anaesthesia practice. Acta Anaesthesiol. Scand..

[32] Ljungqvist O., Scott M., Fearon K.C. (2017). Enhanced Recovery After Surgery: A Review. JAMA Surg..

[33] Deng Q.W., Tan W.C., Zhao B.C., Wen S.H., Shen J.T., Xu M. (2018). Is goal-directed fluid therapy based on dynamic variables alone sufficient to improve clinical outcomes among patients undergoing surgery? A meta-analysis. Crit. Care.

[34] Jacob M., Saller T., Chappell D., Rehm M., Welsch U., Becker B.F. (2013). Physiological levels of A-, B- and C-type natriuretic peptide shed the endothelial glycocalyx and enhance vascular permeability. Basic Res. Cardiol..

[35] Bellomo R. (2014). Issue and challenges of fluid removal in the critically ill. Br. J. Anaesth..

[36] Hahn R.G. (2017). Adverse effects of crystalloid and colloid fluids. Anaesthesiol. Intensive Ther..

[37] Varadhan K.K., Lobo D.N. (2010). A meta-analysis of randomised controlled trials of intravenous fluid therapy in major elective open abdominal surgery: Getting the balance right. Proc. Nutr. Soc..

[38] Li Y., He R., Ying X., Hahn R.G. (2015). Ringer’s lactate, but not hydroxyethyl starch, prolongs the food intolerance time after major abdominal surgery; an open-labelled clinical trial. BMC Anesthesiol..

[39] Alsous F., Khamiees M., DeGirolamo A., Amoateng-Adjepong Y., Manthous C.A. (2000). Negative fluid balance predicts survival in patients with septic shock: A retrospective pilot study. Chest.

[40] Malbrain M.L., Marik P.E., Witters I., Cordemans C., Kirkpatrick A.W., Roberts D.J., Van Regenmortel N. (2014). Fluid overload, de-resuscitation, and outcomes in critically ill or injured patients: A systematic review with suggestions for clinical practice. Anaesthesiol. Intensive Ther..

[41] Wang L., Qiu C., Guan X., Chen M., Chen J., Si X., Du Z., Liu Y., Ouyang B. (2018). Fluid Removal with Ultrasound Guided Protocol Improves the Efficacy and Safety of Dehydration in Post-Resuscitated Critically Ill Patients: A Quasi-Experimental, Before and After Study. Shock.

[42] Cordemans C., De Laet I., Van Regenmortel N., Schoonheydt K., Dits H., Huber W., Malbrain M.L. (2012). Fluid management in critically ill patients: The role of extravascular lung water, abdominal hypertension, capillary leak, and fluid balance. Ann. Intensive Care.

[43] Chaudhry T.Z., Yadav M., Bokhari S.F.H., Fatimah S.R., Rehman A., Kamran M., Asim A., Elhefyan M., Yousif O. (2024). Artificial Intelligence and Machine Learning in Predicting Intradialytic Hypotension in Hemodialysis Patients: A Systematic Review. Cureus.

[44] Sandys V., Sexton D., O’Seaghdha C. (2022). Artificial intelligence and digital health for volume maintenance in hemodialysis patients. Hemodial. Int..

[45] Reljin N., Posada-Quintero H.F., Eaton-Robb C., Binici S., Ensom E., Ding E., Hayes A., Riistama J., Darling C., McManus D. (2020). Machine Learning Model Based on Transthoracic Bioimpedance and Heart Rate Variability for Lung Fluid Accumulation Detection: Prospective Clinical Study. JMIR Med. Inform..

[46] Bateman R.M., Sharpe M.D., Jagger J.E., Ellis C.G. (2015). Sepsis impairs microvascular autoregulation and delays capillary response within hypoxic capillaries. Crit. Care.

[47] Monnet X., Teboul J.L. (2018). My patient has received fluid. How to assess its efficacy and side effects?. Ann. Intensive Care.

[48] Pockevicius V., Cepenas M., Miklusis D., Markevicius V., Zabuliene L., Navikas D., Valinevicius A., Andriukaitis D. (2017). Feasibility research of non-invasive methods for interstitial fluid level measurement. Biomed. Mater. Eng..

[49] O’Doherty J., McNamara P., Clancy N.T., Enfield J.G., Leahy M.J. (2009). Comparison of instruments for investigation of microcirculatory blood flow and red blood cell concentration. J. Biomed. Opt..

[50] Mongkolpun W., Gardette M., Orbegozo D., Vincent J.-L., Creteur J. (2022). An increase in skin blood flow induced by fluid challenge is associated with an increase in oxygen consumption in patients with circulatory shock. J. Crit. Care.

[51] Bezemer R., Lima A., Klijn E., Bakker J., Ince C.J.C.C. (2009). Assessment of tissue oxygen saturation during a vascular occlusion test using near-infrared spectroscopy: Role of the probe spacing and measurement site studied in healthy volunteers. Crit. Care.

[52] Hassan M., Togawa T. (2001). Observation of skin thermal inertia distribution during reactive hyperaemia using a single-hood measurement system. Physiol. Meas..

[53] Vaz P.G., Humeau-Heurtier A., Figueiras E., Correia C., Cardoso J. (2016). Laser Speckle Imaging to Monitor Microvascular Blood Flow: A Review. IEEE Rev. Biomed. Eng..

[54] Aykut G., Veenstra G., Scorcella C., Ince C., Boerma C. (2015). Cytocam-IDF (incident dark field illumination) imaging for bedside monitoring of the microcirculation. Intensive Care Med. Exp..

[55] Veenstra G., Ince C., Barendrecht B.W., Zijlstra H.W., Boerma E.C. (2019). Differences in capillary recruitment between cardiac surgery and septic patients after fluid resuscitation. Microvasc. Res..

[56] Rovas A., Lukasz A.H., Vink H., Urban M., Sackarnd J., Pavenstädt H., Kümpers P. (2018). Bedside analysis of the sublingual microvascular glycocalyx in the emergency room and intensive care unit—The GlycoNurse study. Scand. J. Trauma. Resusc. Emerg. Med..

[57] Pranskunas A., Tamosuitis T., Balciuniene N., Damanskyte D., Sneider E., Vitkauskiene A., Sirvinskas E., Pilvinis V., Boerma E.C. (2018). Alterations of conjunctival glycocalyx and microcirculation in non-septic critically ill patients. Microvasc. Res..

[58] Ostrowski S.R., Haase N., Müller R.B., Møller M.H., Pott F.C., Perner A., Johansson P.I. (2015). Association between biomarkers of endothelial injury and hypocoagulability in patients with severe sepsis: A prospective study. Crit. Care.

[59] Rehm M., Bruegger D., Christ F., Conzen P., Thiel M., Jacob M., Chappell D., Stoeckelhuber M., Welsch U., Reichart B. (2007). Shedding of the endothelial glycocalyx in patients undergoing major vascular surgery with global and regional ischemia. Circulation.

[60] Reitsma S., Slaaf D.W., Vink H., van Zandvoort M.A., oude Egbrink M.G. (2007). The endothelial glycocalyx: Composition, functions, and visualization. Pflug. Arch..

[61] Becker B.F., Chappell D., Bruegger D., Annecke T., Jacob M. (2010). Therapeutic strategies targeting the endothelial glycocalyx: Acute deficits, but great potential. Cardiovasc. Res..

[62] Raper R.F., Cameron G., Walker D., Bowey C.J. (1997). Type B lactic acidosis following cardiopulmonary bypass. Crit. Care Med..

[63] Bahlmann L., Misfeld M., Klaus S., Leptien A., Heringlake M., Schmucker P., Sievers H.H., Ungerstedt U., Kraatz E.G. (2004). Myocardial redox state during coronary artery bypass grafting assessed with microdialysis. Intensive Care Med..

[64] Perz S., Uhlig T., Kohl M., Bredle D.L., Reinhart K., Bauer M., Kortgen A. (2011). Low and “supranormal” central venous oxygen saturation and markers of tissue hypoxia in cardiac surgery patients: A prospective observational study. Intensive Care Med..

[65] Cassina T., Putzu A., Santambrogio L., Villa M., Licker M. (2016). Hemodynamic challenge to early mobilization after cardiac surgery: A pilot study. Ann. Card. Anaesth..

[66] Bienz M., Drullinsky D., Stevens L.M., Bracco D., Noiseux N. (2016). Microcirculatory response during on-pump versus off-pump coronary artery bypass graft surgery. Perfusion.

[67] Levick J.R., Michel C.C. (2010). Microvascular fluid exchange and the revised Starling principle. Cardiovasc. Res..

[68] Reed R.K., Lidén A., Rubin K. (2010). Edema and fluid dynamics in connective tissue remodelling. J. Mol. Cell Cardiol..

[69] Wiig H., Rubin K., Reed R.K. (2003). New and active role of the interstitium in control of interstitial fluid pressure: Potential therapeutic consequences. Acta Anaesthesiol. Scand..

[70] Aukland K., Reed R.K. (1993). Interstitial-lymphatic mechanisms in the control of extracellular fluid volume. Physiol. Rev..

[71] Wiig H. (2011). Pathophysiology of tissue fluid accumulation in inflammation. J. Physiol..

[72] Kumar Y., Dogra A., Kaushik A., Kumar S. (2022). Progressive evaluation in spectroscopic sensors for non-invasive blood haemoglobin analysis—A review. Physiol. Meas..

[73] Akter S., Hossain M.G., Nishidate I., Hazama H., Awazu K. (2018). Medical applications of reflectance spectroscopy in the diffusive and sub-diffusive regimes. J. Near Infrared Spectrosc..

[74] Kavsaoğlu A.R., Polat K., Hariharan M. (2015). Non-invasive prediction of hemoglobin level using machine learning techniques with the PPG signal’s characteristics features. Appl. Soft Comput..

[75] Convertino V.A., Techentin R.W., Poole R.J., Dacy A.C., Carlson A.N., Cardin S., Haider C.R., Holmes III D.R., Wiggins C.C., Joyner M.J. (2022). AI-Enabled Advanced Development for Assessing Low Circulating Blood Volume for Emergency Medical Care: Comparison of Compensatory Reserve Machine-Learning Algorithms. Sensors.

[76] Masimo W1® Medical Watch. https://www.masimo.com/products/monitors/masimo-w1-medical-watch/.

[77] Gidado I.M., Qassem M., Triantis I.F., Kyriacou P.A. (2022). Review of Advances in the Measurement of Skin Hydration Based on Sensing of Optical and Electrical Tissue Properties. Sensors.

[78] Zonios G., Bykowski J., Kollias N. (2001). Skin Melanin, Hemoglobin, and Light Scattering Properties can be Quantitatively Assessed *In Vivo* Using Diffuse Reflectance Spectroscopy. J. Investig. Dermatol..

[79] Mamouei M., Chatterjee S., Razban M., Qassem M., Kyriacou P.A. (2021). Design and Analysis of a Continuous and Non-Invasive Multi-Wavelength Optical Sensor for Measurement of Dermal Water Content. Sensors.

[80] Gidado I.M., Nwokoye I.I., Triantis I.F., Qassem M., Kyriacou P.A. (2024). Multi-Modal Spectroscopic Assessment of Skin Hydration. Sensors.

[81] Øien A.H., Justad S.R., Tenstad O., Wiig H. (2013). Effects of hydration on steric and electric charge-induced interstitial volume exclusion--a model. Biophys. J..

[82] Baselet B., Rombouts C., Benotmane A.M., Baatout S., Aerts A. (2016). Cardiovascular diseases related to ionizing radiation: The risk of low-dose exposure (Review). Int. J. Mol. Med..

[83] Vernice N.A., Meydan C., Afshinnekoo E., Mason C.E. (2020). Long-term spaceflight and the cardiovascular system. Precis. Clin. Med..

[84] Patel S. (2020). The effects of microgravity and space radiation on cardiovascular health: From low-Earth orbit and beyond. IJC Heart Vasc..

